# Subjective and objective levels of physical activity and their association with cardiorespiratory fitness in rheumatoid arthritis patients

**DOI:** 10.1186/s13075-015-0584-7

**Published:** 2015-03-13

**Authors:** Chen-an Yu, Peter C Rouse, Jet JCS Veldhuijzen Van Zanten, Nikos Ntoumanis, George D Kitas, Joan L Duda, George S Metsios

**Affiliations:** School of Sport, Exercise and Rehabilitation Sciences, University of Birmingham, Birmingham, UK; Department for Health, University of Bath, Bath, UK; Department of Rheumatology, Dudley Group of Hospitals NHS Trust, Russells Hall Hospital, Dudley, West Midlands UK; Department of Physical Activity, Exercise and Health, University of Wolverhampton, Walsall, West Midlands UK; Health Psychology & Behavioural Medicine Research Group, School of Psychology & Speech Pathology, Curtin University, Perth, Western Australia

## Abstract

**Introduction:**

The aims of the present study were: (a) to examine the agreement between subjective (assessed via the International Physical Activity Questionnaire; IPAQ) and objective (accelerometry; GT3X) physical activity (PA) levels in patients with rheumatoid arthritis (RA), and (b) to evaluate the associations of RA patients’ subjective and objective PA to their scores on the maximal oxygen uptake test (VO2max).

**Methods:**

The participants wore the GT3X for seven days before completing the IPAQ and VO2max test. The Bland-Altman plot was used to illustrate the agreement between the objective and subjective PA data, and the Wilcoxon test was employed to examine the differences. The association between the PA measurement and VO2max test was examined via the correlations and the magnitude was presented by the Steiger’s Z value.

**Results:**

Sixty-eight RA patients (age = 55 ± 13 years, body mass index: 27.8 ± 5.4 kg/m2, median of disease duration = 5 (2–8) yrs) were recruited. Smaller differences between the subjective and objective measures were found when PA was assessed at the moderate level. Wilcoxon tests revealed that patients reported less time spent engaged in sedentary behaviours (Z = −6.80, *P* < 0.01) and light PA (Z = −6.89, *P* < 0.01) and more moderate PA (Z = −6.26, *P* < 0.01) than was objectively indicated. Significant positive correlations were revealed between VO2max with all PA levels derived from accelerometry (light PA rho = .35, *P* < .01; moderate PA rho = .34, *P* = .01; moderate and vigorous PA, (MVPA) rho = .33, *P* = .01), and a negative association to sedentary time (ST) emerged (rho = −.27, *P* = .04). IPAQ-reported moderate PA and MVPA positively correlated with maxV02 (rho = .25, *P* = .01, rho = .27, *P* = .01, respectively). Differences between the magnitude of correlations between the IPAQ-VO2 max and GT3X-VO2 max were only significant for ST (Z = 3.43, *P* < .01).

**Conclusions:**

Via responses to the IPAQ, RA patients reported that they were less sedentary and engaged in more higher intensity PA than what was objectively assessed. Accelerometry data correlated with VO2max at all PA levels. Only subjective moderate and MPVA correlated with VO2max. Findings suggest that self-reported PA and ST should be interpreted with caution in people with RA and complemented with accelerometry when possible.

**Trial registration:**

Trial registration: ClinicalTrials.gov ISRCTN04121489. Registered 5 September 2012.

## Introduction

Rheumatoid arthritis (RA), the most common inflammatory musculoskeletal disease, is characterized by joint swelling, pain and bone destruction but also a greater risk of cardiovascular disease (CVD) [1,2]. The latter has been partly attributed to an increased prevalence of classical CVD risk factors [3-5] and the effects of high-grade inflammation on the vasculature [6,7]. Another important factor that may lead to an increased CVD risk in RA is low levels of physical activity (PA) [8,9]. RA patients can and should engage in PA, as exercise may slow down disease progression and improve physical ability [10]. Nevertheless, it is repeatedly shown that PA levels are significantly lower in RA compared to the general population [11-14]. It seems, therefore, important that accurate methods should be available to both evaluate and monitor PA levels in RA patients.

There are two ways of measuring PA, namely subjective and the objective methods, which are distinguished on how the data are collected. To date, subjective PA is predominantly measured via self-reported means, given that this method is easy to administer, low cost, and more efficient at gathering data from larger samples [15]. However, self-reported PA is subject to different types of bias [16] due to lack of understanding and/or differential perceptions of item content when employed in different populations. Due to increased physical disability there may be a risk of RA patients to over-report their PA. Further, it is not clear how self- reported PA corresponds to indicators of physical function in RA patients. On the other hand, accelerometry is one of the most frequent ways of measuring objective PA, as the device is light to carry and non-invasive [17]. Although the accelerometer is not the most accurate assessment of objective PA assessment, data obtained from accelerometry are generated in real time and have been validated [18] in both field and lab settings [19]. Increased PA is a behaviour that results in physiological adaptations that, in turn, may prevent or improve disease-related factors and CVD risk in RA patients [11,20]. This beneficial association is strongly supported by a robust inverse relationship of CVD morbidity and mortality with cardiorespiratory fitness, assessed via the ‘gold standard’ method, namely, the maximal oxygen uptake (VO2max) test [21,22]. Therefore, the aims of the present study were to: 1) examine the agreement between subjective (questionnaire) and objective (accelerometry) levels of PA in RA patients, and 2) investigate the associations of these two assessments against VO2max.

## Methods

### Participants and procedure

Sixty eight RA patients (age = 55 ± 13 years, body mass index: 27.8 ± 5.4 kg/m^2^, median of disease duration = five (two to eight) years) participated in this study. The current medication of all studied participants appears in Table 1. The data presented herein are part of a randomised controlled trial study with 100 participants (register number: ISRCTN04121489); in this study we present the baseline data from the participants who provided complete data for objective and subjective PA as well as VO2max data (68 out of 100). All patients fulfilled the revised American College of Rheumatology classification criteria for RA [23] and were recruited from Russells Hall Hospital (Dudley Group NHS Foundation Trust, UK). The study protocol and the main trial were approved by Birmingham East, North and Solihull Research Ethics Committee. All participants provided informed consent after verbal and written information was presented to them about all procedures involved in the project. Each participant visited the laboratory (Clinical Research Unit) on two separate occasions, seven days apart. During the first visit, participants were provided with a questionnaire pack, including the Health Assessment Questionnaire (HAQ) [24] to complete at a time convenient for themselves. During the same visit, an accelerometer (GT3X, ActiGraph, Pensacola, FL, USA) was provided to the participants to be worn over the subsequent seven consecutive days in order to assess objective PA. In addition, clinical disease activity and physical function were assessed as described below. During the second visit, participants returned the accelerometer and questionnaire pack and completed an assisted long form of the International Physical Activity Questionnaire (IPAQ) [15], in order to evaluate their subjective PA levels. This was then followed by the assessment of cardiorespiratory fitness, with a VO2max test.

**Table 1:** **Demographic characteristics and medication of RA patients**

**Demographic characteristics**	**Mean±SD/Mean (%)**
Age, mean ± SD years	55 ± 13
Women (%)	42 (62%)
Height, mean ± SD	168.2 ± 9.3
BMI, mean ± SD years	27.8 ± 5.4
Disease duration, mean ± SD years	7.2 ± 8.7
Medication Number (%)	
Methotrexate	50 (74)
Other DMARDs	35 (52)
Anti-TNF Therapy	8 (12)
Other Biologics	2 (3)
Prednisolone	11 (16)
NSAIDs	24 (35)
Analgesics	25 (37)
Cholesterol-reducing	18 (27)
Anti-Hypertensives	16 (24)

BMI, body mass index; DMARDs, disease-modifying anti-rheumatic drugs; NSAIDs, non-steroidal anti-inflammatory drugs; RA, rheumatoid arthritis.

### Demographic and anthropometric characteristics

Height was measured with a Seca Stadiometer 208 while body mass index and body composition were evaluated via bioelectrical impedance (Tanita BC418-MA, Tokyo, Japan).

### Subjective (self-reported) physical activity

Subjective PA was assessed using the long form of the IPAQ [15] which has previously been used in the case of RA patients [19]. The IPAQ measures the level of PA across four domains, that is, leisure time PA, domestic and gardening activities, work-related PA, and transport-related PA. In each domain, the duration (in minutes) and frequency (days) of PA including sitting, walking, moderate and vigorous PA are self-reported.

### Objective physical activity

Daily objective PA and sedentary time (ST) were measured using the ActiGraph accelerometer (GT3X, Pensacola, FL, USA). Accelerometry is widely used for assessing individual levels of daily activity/time spent sedentary across different age groups ranging from children [25] to older people [26], as well as in the case of the general population from different countries [27,28]. The validity and reliability [29] of the GT3X has been examined against a previous accelerometer model [30] and across different testing situations including comparisons between mechanical and real life settings [19].

Movements recorded by the GT3X are converted into ‘counts’ within each epoch. These counts are calculated in relation to time spent at different activity intensities according to different cut-off points [27]. Daily averages were calculated for different activity levels including ST, light, moderate, vigorous and moderate-to-vigorous physical activity (MVPA). The cut-off points applied in the present study stemmed from Troiano and his colleagues’ research involving more than 6,000 participants [27] (Sedentary 0 to 99 counts per minute (CPM), Light 100 to 2,019 CPM, moderate 2,020 to 5,998 CPM and vigorous 5,999 CPM and above, MVPA 2,020 CPM and above). In the present study, the accelerometers were initialized in 60 second epochs replicating previous research protocols involving RA patients [17]. Data were screened and to be included in the analyses, the participant had to have valid data for a minimum of 10 hours per day [13] and for at least four days [31].

Participants in the current study were advised to wear the accelerometer on their waist at all times during a seven-day period, including their sleeping time if they did not find this uncomfortable. It was recommended to the participants that they should only take the device off during showering, bathing or engaging in any other water activities. If the participants engaged in any other water activity which was not possible to record on the accelerometer, they were requested to report the activity type, intensity and duration when they returned the device to the researchers. However, this was not the case for any of the current study participants.

### Assessment of cardiorespiratory fitness (VO2max)

The VO2max test was performed using a breath-by-breath system (Metalyzer 3B, Leipzig, Germany) on a HP Cosmos mechanical treadmill (Nussdorf-Traunstein, Germany). After identifying a convenient speed for each individual patient (which was normally 2 mph), the speed increased by 0.5 mph every minute until it reached 4 mph while inclination remain constant at 1%. Thereafter, there were only increases in inclination by 1% every 30 seconds until volitional exhaustion.

### Data analysis

Normal distribution of the variables was assessed via the Kolmogorov-Smirnov normality test. To test our first hypothesis, Bland-Altman plots [32] were used to evaluate the agreement between the different methods of assessing PA (that is, subjective versus objective). Furthermore, given the non-normal distribution of both the subjective (IPAQ) and objective (GT3X) PA data, the Wilcoxon signed-rank test was used to test differences between these two methods for ST, light, moderate and MVPA levels.

To test our second hypothesis, correlations between VO2max with subjective and objective PA at different levels were examined via Spearman correlations. Furthermore, Steiger’s Z test [33] was used for analysing the differences between the correlations of subjective PA versus VO2max, and objective PA versus VO2max at ST, light, moderate, vigorous and MVPA levels. All statistical analyses were conducted using SPSS version 21 for windows package (SPSS, Chicago, IL, USA) with the level of significance set at *P* <0.05.

## Results

Patient characteristics are reported in Table 1.

### Research hypothesis 1: agreement between objective and subjective physical activity

#### The Bland-Altman plots

**Figure 1:**
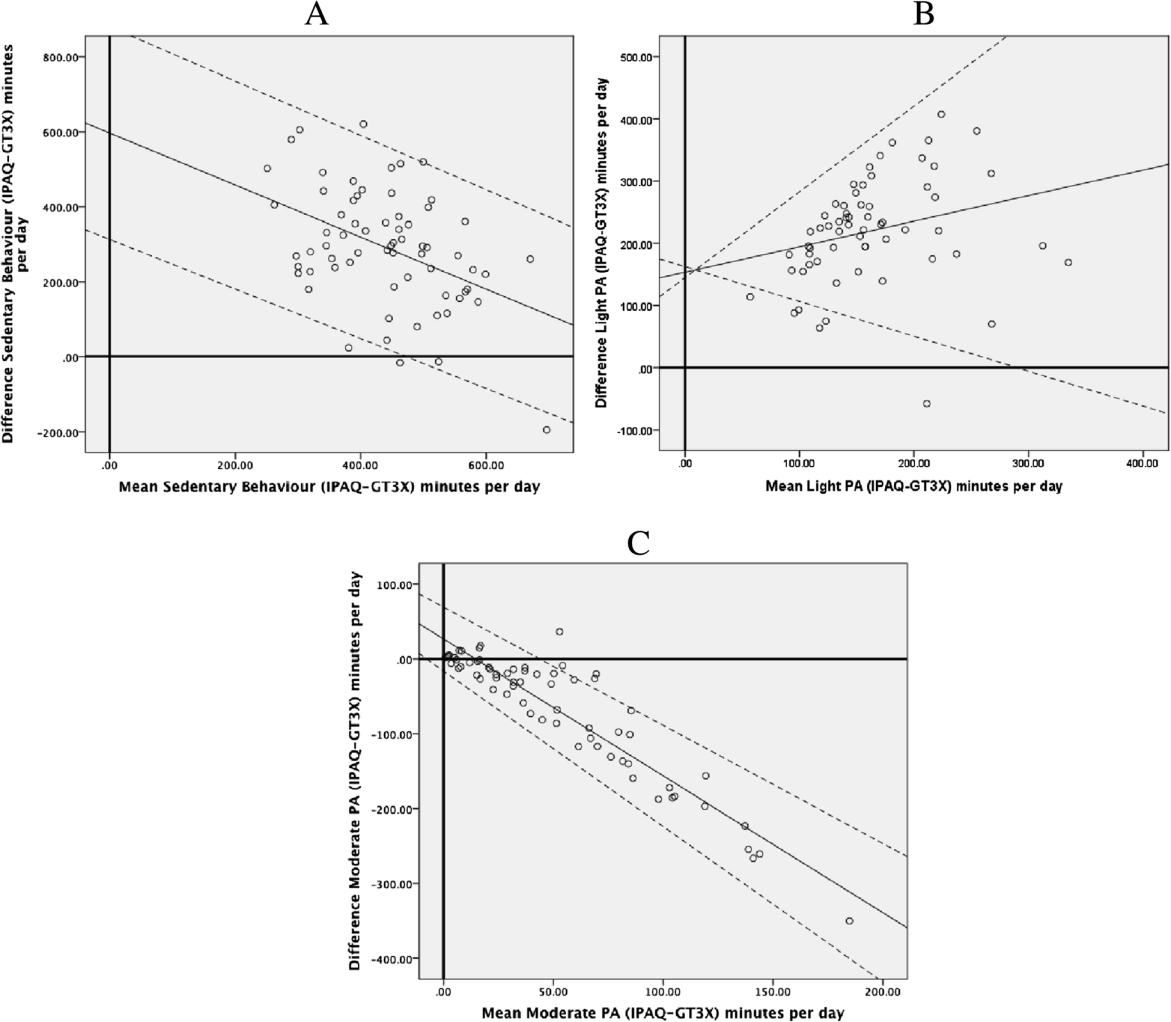
**Bland-Altman plots for minutes per day reported for different level of PA from the GT3X and IPAQ. A)** Sedentary level PA. **B)** Light level PA. **C)** Moderate level PA. IPAQ, International Physical Activity Questionnaire; PA, physical activity.

Separate Bland-Altman plots were produced for ST, light PA, and moderate PA (Figure 1). Results indicated that the degree of agreement between the different methods of assessment varies depending on the level of PA targeted. Smaller differences between the subjective and objective measures emerged when PA was assessed at the moderate level.

Figures 1A and B demonstrated that the mean differences between subjective and objective data for sedentary behaviour time and light PA were spread evenly in the graph. Assessment of the Bland-Altman plots for moderate PA in Figure 1C, however, revealed that at higher levels of PA, the difference between subjective and objective PA becomes greater. In these cases, the subjectively-reported levels were greater than what was revealed via objective GT3X data. Due to the limited observations from the GT3X at the vigorous level, it was not possible to ascertain the pattern of differences between the two measurements from the Bland-Altman plots for this level of activity.

### Differences between subjective (IPAQ) and objective (GT3X) physical activity

The results of the Wilcoxon tests between subjective and objective PA data at different PA levels and the interquartile ranges are shown in Table 2. Self-reported sitting (median (interquartile range) = 337 (225 to 451) minutes) was significantly less than objectively measured ST (median = 596 (510 to 651) minutes; *Z* = −6.80, *P* <0.01). In addition, self-reported light PA (median = 30 (11 to 66) minutes) was also significantly less compared to objective light PA time (median = 267 (221 to 321) minutes; *Z* = −6.89, *P* <0.01). In contrast, participants self-reported significantly more moderate PA behaviour on IPAQ (median = 55 (19 to 129) minutes) than was recorded on GT3X (median = 14 (5 to 25) minutes; *Z* = −6.26, *P* <0.01), and more vigorous PA (median = 0 (0 to 0) minutes) compared to GT3X (median = 0 (0 to 0) minutes, *Z* = −2.83, *P* = 0.01]. Subjective participation in MVPA (median = 59 (22 to 148) minutes) was also significantly elevated compared to MVPA objectively measured via GT3X (median = 14 (6 to 26) minutes; *Z* = −6.28, *P* <0.01).

**Table 2:** **Comparisons between IPAQ and accelerometry data at different physical activity levels and median, interquartile information**

**Physical activity level**	**Accelerometry**	**IPAQ (range 0 to 1440)**	***Z***
Sedentary time (minutes per day)	569 (124 to 765)	337 (82 to 592)	−6.80*
Light PA (minutes per day)	267 (144 to 390)	30 (0 to 87)	−6.89*
Moderate PA (minutes per day)	14 (0 to 34)	55 (0 to 158)	−6.26*
MVPA (minutes per day)	14 (0 to 34)	59 (0 to 181)	−6.28*

Data reported as median ± interquartile range. *P <0.05. IPAQ, International Physical Activity Questionnaire; MVPA, moderate to vigorous physical activity; PA. physical activity.

### Research hypothesis 2: associations between subjective (IPAQ) and objective (GT3X) physical activity with VO2max

The average VO2max was 20.17 ± 4.60 ml/minute/kg, which is indicative of poor fitness levels [11]. Overall, weak associations emerged between VO2max and subjectively reported ST levels, while the correlations between VO2max and objective PA levels were stronger (Table 3). VO2max correlated significantly only with subjective moderate PA (rho = .25, *P* = 0.01) and MVPA (rho = .27, *P* = 0.01) levels. On the other hand, VO2max correlated significantly with all objective GT3X PA measurements (light PA rho = .35, *P* <0.01; moderate PA rho = .34, *P* = 0.01; MVPA rho = .33, *P* = 0.01), whereas objective ST was negatively associated with VO2max fitness (rho = −.27, *P* = 0.04).

**Table 3:** **The mean, standard deviation, interquartile range for and Spearman’s correlations between the study variables**

**Variables**	**M**	**SD**	**IQR**	**(1)**	**(2)**	**(3)**	**(4)**	**(5)**	**(6)**	**(7)**	**(8)**	**(9)**	**(10)**
(1) GT3X sedentary (minutes per day)	583	98	142	-									
(2) GT3X light PA (minutes per day)	275	79	123	-.78**	-								
(3) GT3X moderate PA (minutes per day)	19	17	20	-.44**	.37**	-							
(4) GT3X MVPA (minutes per day)	19	17	20	-.44**	.37**	1**	-						
(5) Step counts	5378	2708	3716	-.64**	.59**	.78**	.78**	-					
(6) IPAQ sitting (minutes per day)	290	159	255	.32*	-.37*	-.15	-.15	-.33**	-				
(7) IPAQ walking (minutes per day)	616	653	57	-.18	.19	.30*	.29*	.21	-.24*	-			
(8) IPAQ moderate PA (minutes per day)	85	275	103	-.22	.25*	.28*	.27*	.28*	-.25*	.42**	-		
(9) IPAQ vigorous PA (minutes per day)	701	785	0	-.09	.08	.01	.01	.08	-.00	.16	.34**	-	
(10) IPAQ MVPA (minutes per day)	510	731	122	-.24	.27*	.28*	.27*	.29*	-.24*	.43**	.98**	.45**	-
(11) VO2 max (ml/min/kg)	20.17	4.60	7.17	-.27*	.35**	.34**	.33*	.48**	-.02	.11	.25*	.19	.27**

IPAQ, International Physical Activity Questionnaire; IQR, interquartile range; M, mean; MVPA, moderate to vigorous physical activity; PA, physical activity; SD, standard deviation. *P <0.05, **P <0.01.

The tests of the magnitude of the differences between correlations were examined according to the results of the Steiger's Z value. Results indicated that only the differences between the correlations for ST (Z = 3.43, *P* <0.001) were significant, whereas at all other levels of PA, no significant differences emerged, that is, light PA (Z = −1.03, *P* = 0.31), moderate PA (Z = −.04, *P* = .67), vigorous PA (Z = −1.10, *P* = 0.27) and MVPA (Z = −0.43, *P* = .67).

## Discussion

The present study examined in RA patients: 1) the agreement between subjective/self-reported (IPAQ) and objective (GT3X) PA, and 2) the associations between both of these assessments with VO2max, the gold standard assessment of cardiorespiratory fitness. Our results show that, when compared to accelerometry-derived values, RA patients under-reported ST while in contrast, they over-reported moderate PA, vigorous PA and MVPA when these behaviours are assessed using a self-report questionnaire. In addition, we found VO2max to associate more with objective, in contrast to subjective, PA.

Based on the data obtained from the GT3X, the present findings indicated that this sample of RA patients had both lower PA compared to relevant guidelines of 150 minutes of moderate intensity PA or 75 minutes of vigorous PA per week (or an equivalent mix of both) [34]. Our results echo past work [35] that assessed PA (via the QUEST-RA) in people with RA conducted in different countries, which revealed only a minority of RA patients (13.8%) exercise more than three times a week. Other research has revealed that even if RA patients are aware of the guidelines for PA [36], they generally do not reach recommended levels of PA. As such, identifying barriers and facilitators of PA amongst RA patients is certainly an area warranting future research.

Examination of the agreement between both subjective and objective PA measurements indicated that the two measurements varied in their degree of discrepancy at the different PA levels. The Bland-Altman plots for sedentary and light PA levels revealed lower agreement between these two measurements when RA patients were less active. Similar patterns have been identified in past work in the general population [37]; however, the lack of agreement was even larger in the current study as well as when compared to research on another patient group, that is, hip osteoarthritis (OA) patients [38]. Even more pronounced than what was the case for hip OA patients, PA was over reported in our sample of RA patients when contrasted to what was observed via objective PA measurement. The accelerometry-derived data in the current study were found to have higher agreement with the gold standard (VO2max) compared to previous research on RA patients involving objective assessments of PA [39]. In the study by Backhouse and colleagues [39], however, objective PA was assessed during the completion of a walking task. Participants in the current study were requested to wear the accelerometer over a seven-day period and, thus, were likely to be engaging in a variety of activities including sedentary behaviours.

In regards to comparing self-reported levels of PA with the gold standard measure of fitness (VO2max), only moderate PA and MVPA were significantly correlated with VO2max. In contrast, all objectively assessed PA levels as well as time spent sedentary were significantly associated with cardiorespiratory fitness in the expected directions. These results suggest that increased engagement in objectively assessed MVPA may lead to increased cardiorespiratory fitness in the RA population. Increased cardiorespiratory fitness is important to enhanced cardiovascular health in RA patients [7,20], as has also been shown in research on coronary artery disease patients [40].

Despite the implications of our findings, it is equally important to acknowledge some of the limitations of the current study. The sample size was not calculated using standardized techniques and was limited to a certain geographical area within the UK. Also, previous research [41] has indicated that the over-reporting of PA is usually found in certain groups such as overweight people, which was indeed the case for our participants (BMI of >28). However, it is important to point out that overweight or obese states are highly prevalent in the RA population [42-44].

## Conclusion

Overall, our findings suggest that the observed associations highlighted a discrepancy between RA patients’ perceptions of their participation in PA and ST (as determined via responses to the IPAQ) and what was the case objectively. Patients with RA self-reported significantly less engagement in sedentary pursuits and significantly more moderate and vigorous PA than what were indicated via accelerometer assessments. Moreover, the associations between IPAQ-assessed sitting time and PA levels and the gold standard assessment of PA indicated that only self-reported moderate and MPVA were correlated with maxVO2 in the expected manner. The present findings suggest that results from existing studies of RA patients’ PA and sedentary behaviour patterns that are based solely on subjective measures of PA and sedentary behaviour (such as the IPAQ) should be interpreted with caution.

## Abbreviations

BMI: body mass index kg/m^2^
CPM: counts per minute
CVD: cardiovascular disease
HAQ: Health Assessment Questionnaire
IPAQ: The long form of the International Physical Activity Questionnaire
MVPA: moderate to vigorous physical activity
PA: physical activity
RA: rheumatoid arthritis
ST: sedentary time, the time that has been spend on the behaviours remains at sedentary level

## References

[1] Crowson CS, Liao KP, Davis JM, Solomon DH, Matteson EL, Knutson KL (2013). Rheumatoid arthritis and cardiovascular disease. Am Heart J..

[2] Kitas GD, Gabriel SE (2010). Cardiovascular disease in rheumatoid arthritis state of the art and future perspectives. Ann Rheum Dis..

[3] Desseing PH, Joffe BI (2006). Insulin resistance and impaired beta cell function in rheumatoid arthritis. Ann Rheum Dis..

[4] Gonzalez-Gay MA, Llorca J, Garcia-Unzueta MT, Gonzalez-Juanatey C, De Matias JM, Martin J (2008). High-grade inflammation, circulating adiponectin concentrations and cardiovascular risk factors in severe rheumatoid arthritis. Clin Exp Rheumatol..

[5] Panoulas VF, Metsios GS, Pace AV, John H, Treharne GJ, Banks MJ (2008). Hypertension in rheumatoid arthritis. Rheumatology..

[6] Sandoo A, van Zanten JJ V, Metsios GS, Carroll D, Kitas GD (2011). Vascular function and morphology in rheumatoid arthritis: a systematic review. Rheumatology..

[7] Metsios GS, Stavropoulos-Kalinoglou A, van Zanten JJ V, Nightingale P, Sandoo A, Dimitroulas T (2013). Individualised exercise improves endothelial function in patients with rheumatoid arthritis. Ann Rheum Dis..

[8] Metsios GS, Stavropoulos-Kalinoglou A, Panoulas VF, Wilson M, Nevill AM, Koutedakis Y (2009). Association of physical inactivity with increased cardiovascular risk in patients with rheumatoid arthritis. Eur J Cardiovasc Prev Rehabil..

[9] Metsios GS, Stavropoulos-Kalinoglou A, Sandoo A, Velduijzen van Zanten JJ, Toms TE, John H (2010). Vascular function and inflammation in rheumatoid arthritis: the role of physcial activity. Open Cardiovasc Med J..

[10] de Jong Z, Munneke M, Zwinderman AH, Kroon HM, Ronday KH, Lems WF (2004). Long term high intensity exercise and damage of small joints in rheumatoid arthritis. Ann Rheum Dis..

[11] Metsios GS, Stavropoulos-Kalinoglou A, Veldhuijzen van Zanten JJ, Treharne GJ, Panoulas VF, Douglas KM (2007). Rheumatoid arthritis, cardiovascular disease and physical exercise: a systematic review. Rheumatology..

[12] Tierney M, Fraser A, Kennedy N (2012). Physical activity in rheumatoid arthritis: a systematic review. J Phys Act Health..

[13] Prioreschi A, Hodkinson B, Avidon I, Tikly M, McVeigh JA (2013). The clinical utility of accelerometry in patients with rheumatoid arthritis. Rheumatology..

[14] Lee J, Dunlop D, Ehrlich-Jones L, Semanik P, Song J, Manheim L (2012). Public health impact of risk factors for physical inactivity in adults with rheumatoid arthritis. Arthritis Care Res..

[15] Craig CL, Marshall AL, Sjöstöm M, Bauman AE, Booth ML, Ainsworth BE (2003). International physical activity questionnaire: 12-country reliability and validity. Med Sci Sports Exerc..

[16] Eurenius E, Stenstrom CH (2005). Physical activity, physical fitness, and general health perception among individuals with rheumatoid arthritis. Arthritis Rheum..

[17] Semanik P, Song J, Chang RW, Manheim L, Ainsworth B, Dunlop D (2010). Assessing physical activity in persons with rheumatoid arthritis using accelerometry. Med Sci Sports Exerc..

[18] Henelman D, Miller K, Baggett C, Debold E, Freedson P (2000). Validity of accelerometry for the assessment of moderate intensity physical activity in the field. Med Sci Sports Exerc.

[19] Ried-Larsen M, Brønd JC, Brage S, Hansen BH, Grydeland M, Andersen LB (2012). Mechanical and free living comparisons of four generations of the Actigraph activity monitor. Int J Behav Nutr Phys Act..

[20] Stavropoulos-Kalinoglou A, Metsios GS, Veldhuijzen van Zanten JJ, Nightingale P, Kitas GD, Koutedakis Y (2012). Individualised aerobic and resistance exercise training improves cardiorespiratory fitness and reduces cardiovascular risk in patients with rheumatoid arthritis. Ann Rheum Dis..

[21] Sui X, LaMonte MJ, Laditka JN, Hardin JW, Chase N, Hooker SP (2007). Cardiorespiratory fitness and adiposity as mortality predictors in older adults. JAMA..

[22] Myers J, Prakash M, Froelicher V, Do D, Partington S, Atwood JE (2002). Exercise capacity and mortality among men referred for exercise testing. N Engl J Med..

[23] Arnett FC, Edworthy SM, Bloch DA, McShane DJ, Fries JF, Cooper NS (1988). The American Rheumatism Association 1987 revised criteria for the classification of rheumatoid arthritis. Arthritis Rheum..

[24] Kirwan JR, Reeback JS (1986). Stanford Health Assessment Questionnaire modified to assess disability in British patients with rheumatoid arthritis. Br J Rheumatol..

[25] Kwon S, Janz KF (2012). Tracking of accelerometry-measured physical activity during childhood: ICAD pooled analysis. Int J Behav Nutr Phys Act..

[26] Hutto B, Howard VJ, Blair SN, Colabianchi N, Vena JE, Rhodes D (2013). Identifying accelerometer nonwear and wear time in older adults. Int J Behav Nutr Phys Act..

[27] Troiano RP, Berrigan D, Dodd KW, Masse LC, Tilert T, McDowell M (2007). Physical activity in the United States measured by accelerometer. Med Sci Sports Exerc..

[28] Colley RC, Garriguet D, Janssen I, Craig CL, Clarke J, Tremblay MS (2011). Physical activity of Canadian adults: accelerometer results from the 2007 to 2009 Canadian Health Measures Survey. Health Rep..

[29] Santos-Lozano A, Santin-Medeiros F, Cardon G, Torres-Luque G, Bailon R, Bergmeir C (2013). Actigraph GT3X: validation and determination of physical activity intensity cut points. Int J Sports Med..

[30] Sasaki JE, John D, Freedson PS (2011). Validation and comparison of ActiGraph activity monitors. J Sci Med Sport..

[31] Trost SG, McIver KL, Pate RR (2005). Conducting accelerometer-based activity assessments in field-based research. Med Sci Sports Exerc..

[32] Bland JM, Altman DG (1986). Statistical methods for assessing agreement between two methods of clinical measurement. Lancet..

[33] Steiger JH (1980). Tests for comparing elements of a correlation matrix. Psychol Bull..

[34] Haskell WL, Lee IM, Pate RR, Powell KE, Blair SN, Franklin BA (2007). Physical activity and public health: updated recommendation for adults from the American College of Sports Medicine and the American Heart Association. Med Sci Sports Exerc..

[35] Sokka T, Häkkinen A, Kautiainen H, Maillefert JF, Toloza S, Mork Hansen T (2008). Physical inactivity in patients with rheumatoid arthritis: Data from twenty-one countries in a cross-sectional, international study. Arthritis Rheum..

[36] Law RJ, Markland DA, Jones JG, Maddison PJ, Thom JM (2013). Perceptions of issues relating to exercise and joint health in rheumatoid arthritis: a UK-based questionnaire study. Musculoskeletal Care..

[37] Hagstromer M, Ainsworth BE, Oja P, Sjostrom M (2010). Comparison of a subjective and an objective measure of physical activity in a population sample. J Phys Act Health..

[38] Svege I, Kolle E, Risberg MA (2012). Reliability and validity of the Physical Activity Scale for the Elderly (PASE) in patients with hip osteoarthritis. BMC Musculoskelet Disord..

[39] Backhouse MR, Hensor EM, White D, Keenan AM, Helliwell PS, Redmond AC (2013). Concurrent validation of activity monitors in patients with rheumatoid arthritis. Clin Biomech (Bristol, Avon).

[40] Ekelund U, Tingström P, Kamwendo K, Krantz M, Nylander E, Sjöström M (2002). The validity of the computer science and applications activity monitor for use in coronary artery disease patient during level walking. Clin Physiol Funct Imaging..

[41] Slootmaker SM, Schuit AJ, Chinapaw MJ, Seidell JC, van Mechelen W (2009). Disagreement in physical activity assessed by accelerometer and self-report in subgroups of age, gender, education and weight status. Int J Behav Nutr Phys Act..

[42] Stavropoulos-Kalinoglou A, Metsios GS, Koutedakis Y, Kitas GD (2010). Obesity in rheumatoid arthritis. Rheumatology..

[43] Summers GD, Metsios GS, Stavropoulos-Kalinoglou A, Kitas GD (2010). Rheumatoid cachexia and cardiovascular disease. Nat Rev Rheumatol..

[44] Crowson CS, Matteson EL, Davis JM, Gabriel SE (2013). Contribution of obesity to the rise in incidence of rheumatoid arthritis. Arthritis Care Res..

